# Supplementary material for: Augmentation of progestin signaling rescues testis organization and spermatogenesis in zebrafish with the depletion of androgen signaling
Source: eLife. 2022 Feb 28;11:e66118. doi: 10.7554/eLife.66118 (PMC8912926; doi:10.7554/eLife.66118)
Supplement: Supplementary file 1. — (a) The gene list and basemean of the total of 148 differentially expressed genes in the overlapped region of the Venn diagram. (b) The gene list and basemean of the total of 111 differentially expressed genes in the overlapped region of the Venn diagram. [file elife-66118-supp1.docx]

**Supplementary File 1**

**Supplementary File 1a.** The gene list and basemean of the total of 148 differentially expressed genes in the overlapped region of the Venn diagram.

| Gene ID | Name | Con | *cyp17a1*-/- | *cyp17a1*-/-;*ar*-/- | *cyp17a1*-/-;*ar*-/-;*npgr*-/- |
| --- | --- | --- | --- | --- | --- |
| ENSDARG00000071095 | *abi3bpb* | 83.78 | 46.47 | 53.21 | 349.48 |
| ENSDARG00000045180 | *acta2* | 5439.52 | 1621.49 | 812.51 | 78.30 |
| ENSDARG00000001431 | *actn3b* | 671.30 | 647.59 | 554.64 | 253.99 |
| ENSDARG00000103519 | *adamts1* | 28.28 | 31.32 | 48.10 | 262.59 |
| ENSDARG00000061348 | *adat1* | 318.37 | 245.50 | 268.11 | 70.66 |
| ENSDARG00000026764 | *ahsg1* | 672.35 | 1679.08 | 1148.16 | 222.48 |
| ENSDARG00000075067 | *aipl1* | 85.88 | 74.76 | 72.66 | 14.32 |
| ENSDARG00000006546 | *ak4* | 2138.53 | 857.72 | 795.11 | 348.53 |
| ENSDARG00000113858 | *AL928650.4* | 605.32 | 633.44 | 202.62 | 1.00 |
| ENSDARG00000097208 | *alkbh3* | 419.96 | 245.50 | 218.99 | 70.66 |
| ENSDARG00000035859 | *angptl4* | 590.66 | 446.54 | 446.16 | 89.76 |
| ENSDARG00000076320 | *ano9a* | 436.71 | 279.85 | 343.83 | 105.04 |
| ENSDARG00000101324 | *apoa1b* | 28.28 | 6272.81 | 4627.42 | 211.02 |
| ENSDARG00000015866 | *apoa2* | 13.61 | 3264.20 | 2398.65 | 85.94 |
| ENSDARG00000010654 | *arhgap42b* | 273.34 | 145.48 | 196.48 | 37.24 |
| ENSDARG00000114760 | *asip2b* | 474.41 | 177.81 | 262.99 | 59.20 |
| ENSDARG00000001870 | *atp1a1a.4* | 566.57 | 605.16 | 398.07 | 157.55 |
| ENSDARG00000099412 | *bcan* | 42.94 | 84.86 | 56.28 | 507.03 |
| ENSDARG00000109968 | *BX537120.1* | 165.47 | 102.04 | 71.63 | 13.37 |
| ENSDARG00000112176 | *BX537120.2* | 278.57 | 169.73 | 138.15 | 21.96 |
| ENSDARG00000116369 | *BX537120.3* | 515.26 | 314.20 | 266.06 | 82.12 |
| ENSDARG00000039098 | *ca8* | 518.40 | 275.81 | 375.56 | 66.84 |
| ENSDARG00000098248 | *CABZ01081730.1* | 161.28 | 50.51 | 72.66 | 6.68 |
| ENSDARG00000041107 | *cftr* | 1024.23 | 816.30 | 690.74 | 115.54 |
| ENSDARG00000015955 | *cldnc* | 962.44 | 557.67 | 333.60 | 134.64 |
| ENSDARG00000093267 | *cngk* | 15248.25 | 15922.96 | 13442.24 | 5530.57 |
| ENSDARG00000099493 | *CR847906.1* | 33.51 | 57.59 | 25.58 | 1.00 |
| ENSDARG00000007795 | *cst3* | 4093.78 | 3337.95 | 3650.16 | 1732.12 |
| ENSDARG00000013771 | *ctss2.2* | 143.48 | 113.15 | 166.80 | 480.30 |
| ENSDARG00000113786 | *CU896602.3* | 1.00 | 2.02 | 1.02 | 40.10 |
| ENSDARG00000041799 | *cx43* | 2770.03 | 1554.82 | 1986.25 | 548.09 |
| ENSDARG00000055100 | *cxcl12b* | 202.12 | 125.27 | 123.82 | 38.19 |
| ENSDARG00000097556 | *cyp8b2* | 111.01 | 101.03 | 91.07 | 20.05 |
| ENSDARG00000018856 | *dclk1a* | 732.04 | 851.66 | 770.55 | 194.79 |
| ENSDARG00000014626 | *dlx3b* | 26.18 | 19.20 | 10.23 | 119.36 |
| ENSDARG00000092662 | *dnaaf3* | 220.97 | 78.80 | 183.17 | 1.00 |
| ENSDARG00000038788 | *dnai1.2* | 2085.11 | 1646.75 | 1598.42 | 774.39 |
| ENSDARG00000010745 | *dnaja2b* | 959.30 | 781.95 | 612.96 | 192.88 |
| ENSDARG00000004979 | *elovl5* | 275.43 | 470.79 | 298.81 | 80.21 |
| ENSDARG00000015228 | *ero1a* | 175.94 | 173.77 | 201.59 | 494.62 |
| ENSDARG00000014239 | *esyt1b* | 706.91 | 805.19 | 898.47 | 224.39 |
| ENSDARG00000100782 | *f7l* | 401.10 | 272.77 | 303.92 | 82.12 |
| ENSDARG00000061354 | *foxred2* | 210.50 | 293.99 | 326.44 | 66.84 |
| ENSDARG00000104500 | *fyco1a* | 381.21 | 422.30 | 351.00 | 135.59 |
| ENSDARG00000100003 | *glulb* | 2389.87 | 2769.17 | 2834.58 | 963.46 |
| ENSDARG00000043342 | *gpx3* | 705.86 | 436.44 | 572.03 | 81.16 |
| ENSDARG00000075301 | *gsdf* | 16053.60 | 11421.17 | 12444.51 | 4800.10 |
| ENSDARG00000013787 | *gucy1a1* | 401.10 | 413.20 | 377.60 | 82.12 |
| ENSDARG00000003903 | *hapln2* | 67.03 | 48.49 | 40.93 | 1.00 |
| ENSDARG00000027609 | *hpn* | 880.75 | 710.22 | 637.52 | 306.51 |
| ENSDARG00000058365 | *hspb8* | 2050.55 | 1355.79 | 735.76 | 311.29 |
| ENSDARG00000052895 | *htra3a* | 474.41 | 396.03 | 344.86 | 160.42 |
| ENSDARG00000025348 | *igfbp5b* | 793.83 | 501.10 | 663.11 | 133.68 |
| ENSDARG00000040777 | *inhbb* | 618.94 | 588.99 | 759.30 | 1503.91 |
| ENSDARG00000035862 | *insl3* | 2013.90 | 7188.12 | 6295.42 | 19.10 |
| ENSDARG00000098012 | *itgae.1* | 264.96 | 288.94 | 334.62 | 692.28 |
| ENSDARG00000057626 | *josd2* | 1055.65 | 1070.89 | 903.59 | 401.04 |
| ENSDARG00000104665 | *kirrel3l* | 1942.69 | 1437.62 | 1553.39 | 696.10 |
| ENSDARG00000045275 | *klhl14* | 13.61 | 12.12 | 15.35 | 72.57 |
| ENSDARG00000017624 | *krt4* | 1183.41 | 530.40 | 1013.08 | 240.63 |
| ENSDARG00000036830 | *krt91* | 2196.12 | 579.90 | 1038.66 | 201.48 |
| ENSDARG00000104654 | *leap2* | 347.69 | 252.57 | 96.19 | 5.73 |
| ENSDARG00000040528 | *lgals3bpb* | 1120.58 | 1781.12 | 3185.57 | 7628.41 |
| ENSDARG00000068247 | *luzp2* | 99.49 | 67.69 | 100.28 | 11.46 |
| ENSDARG00000063583 | *map2k4a* | 497.45 | 425.33 | 431.84 | 179.51 |
| ENSDARG00000068726 | *masp1* | 397.96 | 322.28 | 270.15 | 105.04 |
| ENSDARG00000053215 | *me1* | 641.98 | 533.43 | 392.95 | 166.15 |
| ENSDARG00000002795 | *meis3* | 194.79 | 190.94 | 328.48 | 1004.52 |
| ENSDARG00000079105 | *mhc2dab* | 613.70 | 795.09 | 103.35 | 2089.24 |
| ENSDARG00000011373 | *mknk2a* | 840.96 | 940.57 | 1252.54 | 270.23 |
| ENSDARG00000033610 | *morn5* | 1672.49 | 2172.09 | 1810.24 | 411.55 |
| ENSDARG00000043093 | *mpeg1.2* | 112.06 | 197.00 | 266.06 | 578.65 |
| ENSDARG00000028740 | *msnb* | 2061.03 | 1888.21 | 2435.49 | 954.86 |
| ENSDARG00000038123 | *myl9a* | 923.69 | 476.85 | 522.91 | 183.33 |
| ENSDARG00000078474 | *myo15ab* | 710.05 | 721.34 | 531.10 | 254.95 |
| ENSDARG00000115421 | *n6amt1* | 134.05 | 110.12 | 119.73 | 380.04 |
| ENSDARG00000011170 | *ndrg2* | 463.94 | 401.08 | 590.45 | 164.24 |
| ENSDARG00000078261 | *nphp3* | 958.25 | 924.40 | 831.95 | 385.76 |
| ENSDARG00000006848 | *parp9* | 796.97 | 367.74 | 568.96 | 1.00 |
| ENSDARG00000052494 | *pcdh18b* | 434.62 | 257.62 | 174.99 | 65.89 |
| ENSDARG00000035966 | *pgr* | 470.22 | 399.06 | 453.33 | 52.52 |
| ENSDARG00000042010 | *pklr* | 695.39 | 1190.11 | 2201.15 | 263.54 |
| ENSDARG00000038941 | *ppp1r1c* | 270.20 | 645.57 | 816.60 | 29.60 |
| ENSDARG00000079274 | *prss59.1* | 7.33 | 42.43 | 45.03 | 146.09 |
| ENSDARG00000031885 | *psmb12* | 138.24 | 51.52 | 105.40 | 554.78 |
| ENSDARG00000001656 | *psmb13a* | 127.77 | 31.32 | 129.96 | 450.70 |
| ENSDARG00000001303 | *psmb8a* | 240.87 | 88.90 | 176.01 | 719.01 |
| ENSDARG00000000656 | *psmb9a* | 152.90 | 35.36 | 111.54 | 392.45 |
| ENSDARG00000021184 | *rbfox1l* | 65.98 | 80.82 | 78.80 | 3.82 |
| ENSDARG00000070038 | *rbp2a* | 272.29 | 147.50 | 165.78 | 19.10 |
| ENSDARG00000019396 | *rergla* | 145.57 | 309.14 | 100.28 | 18.14 |
| ENSDARG00000012506 | *rnpep* | 230.40 | 224.28 | 204.66 | 650.26 |
| ENSDARG00000100188 | *RNPS1* | 388.54 | 215.19 | 98.24 | 16.23 |
| ENSDARG00000036773 | *s100s* | 3.14 | 48.49 | 1.00 | 180.47 |
| ENSDARG00000038574 | *scg2b* | 247.16 | 109.11 | 115.63 | 31.51 |
| ENSDARG00000006307 | *shisa4* | 1919.65 | 1202.23 | 1870.62 | 439.24 |
| ENSDARG00000031745 | *si:busm1-266f07.2* | 852.48 | 808.22 | 210.80 | 2243.93 |
| ENSDARG00000097615 | *si:ch211-108d22.2* | 98.44 | 90.92 | 109.49 | 8.59 |
| ENSDARG00000090930 | *si:ch211-120g10.1* | 109.96 | 66.68 | 49.12 | 1.00 |
| ENSDARG00000094016 | *si:ch211-130h14.4* | 1157.23 | 912.28 | 862.65 | 421.09 |
| ENSDARG00000100771 | *si:ch211-160d14.15* | 532.01 | 272.77 | 81.87 | 1.00 |
| ENSDARG00000089627 | *si:ch211-160d20.5* | 493.26 | 381.88 | 306.99 | 140.36 |
| ENSDARG00000077239 | *si:ch211-165i18.2* | 207.36 | 326.32 | 190.34 | 867.02 |
| ENSDARG00000093284 | *si:ch211-173m16.2* | 244.01 | 132.35 | 406.26 | 1.00 |
| ENSDARG00000071355 | *si:ch211-197g15.7* | 58.65 | 60.62 | 113.59 | 246.35 |
| ENSDARG00000088656 | *si:ch211-212k18.6* | 672.35 | 502.11 | 599.66 | 231.08 |
| ENSDARG00000102593 | *si:ch211-214b16.2* | 141.38 | 229.33 | 40.93 | 1.00 |
| ENSDARG00000074345 | *si:ch211-214b16.4* | 247.16 | 254.59 | 73.68 | 1.00 |
| ENSDARG00000098915 | *si:ch211-216p19.5* | 601.13 | 610.21 | 512.68 | 242.54 |
| ENSDARG00000093748 | *si:ch211-217k17.11* | 179.08 | 168.72 | 332.58 | 811.63 |
| ENSDARG00000101968 | *si:ch211-227p7.1* | 68.07 | 85.87 | 37.86 | 1.00 |
| ENSDARG00000093630 | *si:ch211-249h16.8* | 1036.80 | 1130.50 | 863.68 | 413.46 |
| ENSDARG00000094492 | *si:ch211-284e13.12* | 1.05 | 0.00 | 1.00 | 35.33 |
| ENSDARG00000102856 | *si:ch73-59f11.3* | 51.32 | 38.39 | 57.31 | 2.86 |
| ENSDARG00000087633 | *si:dkey-11o18.5* | 1054.60 | 809.23 | 714.27 | 115.54 |
| ENSDARG00000099428 | *si:dkey-15h8.17* | 130.91 | 271.76 | 258.90 | 1665.28 |
| ENSDARG00000070857 | *si:dkey-32e6.6* | 75.40 | 204.08 | 156.57 | 529.95 |
| ENSDARG00000092361 | *si:dkey-79f11.7* | 229.35 | 222.26 | 146.33 | 48.70 |
| ENSDARG00000095309 | *si:dkey-79f11.9* | 1.00 | 0.00 | 17.40 | 83.07 |
| ENSDARG00000096906 | *si:dkey-7i4.24* | 65.98 | 124.26 | 191.36 | 896.62 |
| ENSDARG00000057262 | *si:dkey-91i10.3* | 676.54 | 303.08 | 237.41 | 77.34 |
| ENSDARG00000095407 | *si:dkey-9i23.8* | 2.09 | 3.03 | 2.05 | 137.50 |
| ENSDARG00000088881 | *si:dkeyp-69b9.3* | 270.20 | 322.28 | 445.14 | 40.10 |
| ENSDARG00000073704 | *si:dkeyp-72g9.4* | 53.41 | 46.47 | 42.98 | 1.00 |
| ENSDARG00000020984 | *slc16a10* | 2695.67 | 1982.16 | 1788.75 | 782.03 |
| ENSDARG00000088891 | *slc23a3* | 42.94 | 47.48 | 41.96 | 2.86 |
| ENSDARG00000054423 | *slc7a6* | 1859.95 | 1278.00 | 1059.13 | 512.76 |
| ENSDARG00000079781 | *slitrk4* | 20.95 | 21.22 | 15.35 | 135.59 |
| ENSDARG00000051763 | *sntb2* | 2186.70 | 1766.97 | 1748.84 | 833.60 |
| ENSDARG00000035459 | *spns3* | 732.04 | 298.03 | 277.32 | 62.07 |
| ENSDARG00000028367 | *sult2st3* | 173.85 | 152.55 | 99.26 | 7.64 |
| ENSDARG00000060711 | *sv2bb* | 1154.09 | 845.60 | 776.69 | 136.55 |
| ENSDARG00000045408 | *tagln* | 3751.32 | 1034.52 | 561.80 | 111.72 |
| ENSDARG00000092774 | *tarsl2* | 116.25 | 165.69 | 76.75 | 427.78 |
| ENSDARG00000042637 | *tert* | 529.92 | 1313.36 | 1116.43 | 220.57 |
| ENSDARG00000097920 | *tex36* | 484.89 | 426.34 | 484.03 | 198.61 |
| ENSDARG00000040249 | *tlr18* | 358.17 | 362.69 | 264.01 | 66.84 |
| ENSDARG00000027129 | *tmem218* | 611.61 | 675.87 | 558.73 | 260.68 |
| ENSDARG00000020890 | *tmod4* | 204.22 | 165.69 | 198.52 | 44.88 |
| ENSDARG00000091221 | *ttc34* | 515.26 | 432.40 | 351.00 | 155.64 |
| ENSDARG00000039522 | *tubb2* | 23.04 | 3.03 | 5.12 | 240.63 |
| ENSDARG00000044400 | *wdr78* | 618.94 | 486.95 | 439.00 | 203.39 |
| ENSDARG00000040159 | *wnt4b* | 30.37 | 53.54 | 100.28 | 232.03 |
| ENSDARG00000110510 | *ybey* | 249.25 | 98.00 | 264.01 | 0.95 |
| ENSDARG00000103947 | *zgc:113295* | 80.64 | 81.83 | 65.49 | 1.00 |
| ENSDARG00000102241 | *zgc:136410* | 45.03 | 40.41 | 134.05 | 443.06 |
| ENSDARG00000078423 | *zgc:194285* | 84.83 | 136.39 | 171.92 | 349.48 |
| ENSDARG00000069796 | *znf1124* | 38.75 | 53.54 | 146.33 | 374.31 |

**Supplementary File 1b.** The gene list and basemean of the total of 111 differentially expressed genes in the overlapped region of the Venn diagram.

| Gene ID | Name | Con | *ar*-/- | *ar*-/-;*npgr*-/- | *cyp17a1*-/-;*ar*-/-;*npgr*-/- |
| --- | --- | --- | --- | --- | --- |
| ENSDARG00000071095 | *abi3bpb* | 85.52 | 649.18 | 555.01 | 349.48 |
| ENSDARG00000045180 | *acta2* | 5552.62 | 830.65 | 465.24 | 78.30 |
| ENSDARG00000005595 | *adgb* | 3598.41 | 1602.36 | 687.08 | 1556.43 |
| ENSDARG00000079983 | *agbl2* | 4065.58 | 1758.58 | 903.73 | 1918.32 |
| ENSDARG00000006546 | *ak4* | 2182.99 | 578.09 | 546.38 | 348.53 |
| ENSDARG00000019001 | *ak7a* | 4062.37 | 1652.88 | 846.76 | 1743.58 |
| ENSDARG00000021913 | *ak9* | 5251.15 | 2213.19 | 1093.63 | 2495.06 |
| ENSDARG00000069518 | *aqp3b* | 227.71 | 10.29 | 6.91 | 15.28 |
| ENSDARG00000010654 | *arhgap42b* | 279.02 | 83.25 | 47.47 | 37.24 |
| ENSDARG00000055128 | *armc4* | 5180.59 | 2300.18 | 1034.07 | 2250.61 |
| ENSDARG00000092067 | *atn1* | 158.22 | 638.89 | 1085.86 | 481.25 |
| ENSDARG00000079453 | *atp8b4* | 1761.79 | 466.77 | 152.78 | 746.70 |
| ENSDARG00000116369 | *BX537120.3* | 525.97 | 103.83 | 118.25 | 82.12 |
| ENSDARG00000011166 | *cahz* | 1643.12 | 387.26 | 403.10 | 513.72 |
| ENSDARG00000053455 | *ccdc103* | 4852.40 | 2157.07 | 857.12 | 2241.07 |
| ENSDARG00000016161 | *ccdc170* | 1479.56 | 620.18 | 304.70 | 567.19 |
| ENSDARG00000027381 | *ccdc171* | 3219.96 | 1071.98 | 482.51 | 773.44 |
| ENSDARG00000077928 | *ccdc173* | 5444.65 | 2426.46 | 1066.87 | 2547.58 |
| ENSDARG00000089320 | *ccdc175* | 3181.48 | 1390.96 | 632.70 | 1315.80 |
| ENSDARG00000032372 | *ccdc180* | 2980.50 | 952.25 | 647.37 | 990.19 |
| ENSDARG00000041107 | *cftr* | 1045.53 | 190.82 | 189.90 | 115.54 |
| ENSDARG00000052764 | *chrnb3a* | 52.38 | 249.76 | 350.44 | 355.21 |
| ENSDARG00000093267 | *cngk* | 15565.29 | 5726.61 | 2024.98 | 5530.57 |
| ENSDARG00000069362 | *CR847571.1* | 174.25 | 24.32 | 28.48 | 23.87 |
| ENSDARG00000102899 | *cremb* | 6542.56 | 2644.42 | 1038.38 | 3109.99 |
| ENSDARG00000030980 | *csrp1b* | 376.30 | 58.00 | 25.89 | 6.68 |
| ENSDARG00000007795 | *cst3* | 4178.90 | 1713.68 | 1032.34 | 1732.12 |
| ENSDARG00000021462 | *daw1* | 9618.20 | 4289.81 | 2113.88 | 3878.66 |
| ENSDARG00000088693 | *dcst2* | 2872.52 | 1218.84 | 707.79 | 1330.13 |
| ENSDARG00000005221 | *desmb* | 427.62 | 14.03 | 31.94 | 16.23 |
| ENSDARG00000075825 | *dlec1* | 4941.13 | 1963.43 | 999.54 | 1904.95 |
| ENSDARG00000012030 | *dnaaf1* | 1915.73 | 782.94 | 378.06 | 846.96 |
| ENSDARG00000100115 | *dnah1* | 10155.93 | 4411.41 | 1503.63 | 4384.73 |
| ENSDARG00000100285 | *dnai2a* | 3487.22 | 1509.76 | 668.09 | 1411.29 |
| ENSDARG00000053498 | *ednrbb* | 3.21 | 80.45 | 215.79 | 792.54 |
| ENSDARG00000004979 | *elovl5* | 281.16 | 43.03 | 36.25 | 80.21 |
| ENSDARG00000070598 | *eno4* | 2892.84 | 1236.62 | 472.15 | 1144.88 |
| ENSDARG00000100426 | *fam167b* | 711.98 | 118.80 | 76.82 | 177.60 |
| ENSDARG00000004177 | *fam169ab* | 109.04 | 502.32 | 690.53 | 308.42 |
| ENSDARG00000001976 | *fkbp16* | 12.83 | 201.11 | 325.41 | 316.06 |
| ENSDARG00000073860 | *gas2l2* | 731.23 | 231.98 | 112.21 | 281.68 |
| ENSDARG00000043342 | *gpx3* | 720.54 | 85.12 | 120.84 | 81.16 |
| ENSDARG00000104686 | *hsf5* | 6674.05 | 2677.16 | 1120.38 | 2912.33 |
| ENSDARG00000058365 | *hspb8* | 2093.19 | 347.97 | 209.75 | 311.29 |
| ENSDARG00000056764 | *hydin* | 4956.09 | 1915.73 | 900.28 | 2351.83 |
| ENSDARG00000090953 | *igsf5b* | 197.77 | 22.45 | 11.22 | 55.38 |
| ENSDARG00000035862 | *insl3* | 2055.77 | 108.51 | 1.73 | 19.10 |
| ENSDARG00000068288 | *lamc2* | 1283.92 | 263.79 | 201.12 | 331.34 |
| ENSDARG00000076153 | *lca5* | 2604.19 | 1110.34 | 589.54 | 1221.27 |
| ENSDARG00000104654 | *leap2* | 354.92 | 1820.32 | 1752.22 | 5.73 |
| ENSDARG00000037278 | *lrata* | 218.09 | 15.90 | 36.25 | 21.96 |
| ENSDARG00000069698 | *lrguk* | 2280.27 | 886.77 | 525.67 | 843.14 |
| ENSDARG00000055839 | *MARCH4* | 3098.09 | 1288.07 | 479.05 | 1287.16 |
| ENSDARG00000079742 | *mcf2l2* | 358.13 | 107.57 | 68.19 | 124.13 |
| ENSDARG00000018008 | *mdh1b* | 2734.62 | 1130.92 | 404.82 | 1014.07 |
| ENSDARG00000002795 | *meis3* | 198.84 | 701.56 | 998.68 | 1004.52 |
| ENSDARG00000059657 | *mks1* | 2500.50 | 920.45 | 510.13 | 972.05 |
| ENSDARG00000104487 | *mthfs* | 2018.36 | 580.89 | 169.18 | 699.92 |
| ENSDARG00000008030 | *myl9b* | 3379.25 | 654.79 | 176.95 | 1459.03 |
| ENSDARG00000061635 | *myo5aa* | 52.38 | 181.47 | 320.23 | 172.83 |
| ENSDARG00000098944 | *nme8* | 3249.90 | 1425.57 | 828.64 | 1440.89 |
| ENSDARG00000042396 | *odf3b* | 3077.78 | 1170.20 | 489.41 | 1338.72 |
| ENSDARG00000038612 | *pih1d3* | 1447.49 | 587.44 | 309.88 | 627.35 |
| ENSDARG00000095386 | *pimr179* | 341.03 | 117.86 | 66.46 | 122.22 |
| ENSDARG00000068190 | *pimr188* | 718.40 | 212.34 | 100.13 | 270.23 |
| ENSDARG00000038941 | *ppp1r1c* | 275.81 | 58.93 | 42.29 | 29.60 |
| ENSDARG00000052121 | *rag2* | 1429.31 | 585.57 | 241.69 | 619.71 |
| ENSDARG00000061985 | *rbm47* | 1884.73 | 425.61 | 273.62 | 865.11 |
| ENSDARG00000023484 | *rflna* | 547.35 | 211.40 | 110.48 | 190.97 |
| ENSDARG00000074828 | *rhobtb2a* | 394.48 | 128.15 | 77.68 | 150.87 |
| ENSDARG00000012504 | *rlbp1a* | 171.05 | 40.22 | 28.48 | 20.05 |
| ENSDARG00000067606 | *rsph4a* | 5282.15 | 2013.95 | 872.66 | 2029.09 |
| ENSDARG00000090767 | *si:ch211-136m16.8* | 45.97 | 290.91 | 333.18 | 209.12 |
| ENSDARG00000053340 | *si:ch211-138g9.3* | 427.62 | 103.83 | 49.20 | 142.27 |
| ENSDARG00000077900 | *si:ch211-141o9.10* | 1530.87 | 489.22 | 195.94 | 514.67 |
| ENSDARG00000079705 | *si:ch211-152p11.4* | 1293.54 | 462.09 | 254.63 | 588.20 |
| ENSDARG00000089627 | *si:ch211-160d20.5* | 503.52 | 121.60 | 82.86 | 140.36 |
| ENSDARG00000098445 | *si:ch211-1o7.2* | 1399.38 | 295.59 | 72.51 | 570.05 |
| ENSDARG00000094491 | *si:ch211-202m22.1* | 503.52 | 179.60 | 75.96 | 187.15 |
| ENSDARG00000093734 | *si:ch211-209a2.2* | 3792.97 | 1488.24 | 647.37 | 1754.08 |
| ENSDARG00000095607 | *si:ch211-209f23.3* | 304.68 | 101.96 | 53.52 | 89.76 |
| ENSDARG00000097680 | *si:ch211-241f5.3* | 423.34 | 69.22 | 80.27 | 47.74 |
| ENSDARG00000093630 | *si:ch211-249h16.8* | 1058.35 | 392.87 | 214.06 | 413.46 |
| ENSDARG00000097256 | *si:ch211-270g19.5* | 492.83 | 56.12 | 34.53 | 68.75 |
| ENSDARG00000102101 | *si:ch73-174h16.5* | 3436.98 | 1528.47 | 816.55 | 1487.68 |
| ENSDARG00000095463 | *si:ch73-90k17.1* | 7.48 | 101.96 | 387.56 | 60.16 |
| ENSDARG00000099207 | *si:dkey-11f4.7* | 26.73 | 162.76 | 222.70 | 189.06 |
| ENSDARG00000087633 | *si:dkey-11o18.5* | 1076.53 | 253.50 | 70.78 | 115.54 |
| ENSDARG00000070511 | *si:dkey-183j2.10* | 58.80 | 1473.28 | 748.36 | 2501.74 |
| ENSDARG00000045094 | *si:dkey-222b8.1* | 375.23 | 82.32 | 53.52 | 108.85 |
| ENSDARG00000092275 | *si:dkey-234d14.2* | 769.71 | 277.82 | 164.00 | 305.56 |
| ENSDARG00000062606 | *si:dkey-26i13.8* | 2445.97 | 1070.11 | 521.35 | 1100.00 |
| ENSDARG00000094514 | *si:dkey-7l6.3* | 571.94 | 182.41 | 119.12 | 227.26 |
| ENSDARG00000057262 | *si:dkey-91i10.3* | 690.60 | 179.60 | 104.44 | 77.34 |
| ENSDARG00000094330 | *si:dkey-9p24.5* | 2876.80 | 1151.50 | 428.99 | 1350.18 |
| ENSDARG00000088881 | *si:dkeyp-69b9.3* | 275.81 | 71.09 | 27.62 | 40.10 |
| ENSDARG00000093322 | *si:dkeyp-87a6.2* | 357.06 | 116.93 | 52.65 | 134.64 |
| ENSDARG00000105383 | *si:zfos-405g10.4* | 2223.61 | 719.33 | 214.93 | 911.89 |
| ENSDARG00000104573 | *slc12a2* | 3978.99 | 1114.08 | 828.64 | 1730.21 |
| ENSDARG00000020984 | *slc16a10* | 2751.72 | 850.29 | 574.87 | 782.03 |
| ENSDARG00000090106 | *slc17a7b* | 256.57 | 11.22 | 9.49 | 11.46 |
| ENSDARG00000003697 | *slc5a8l* | 409.44 | 72.96 | 50.93 | 47.74 |
| ENSDARG00000005894 | *slc7a9* | 377.37 | 64.54 | 60.42 | 56.34 |
| ENSDARG00000075015 | *soul5* | 152.87 | 506.99 | 1355.16 | 1941.24 |
| ENSDARG00000035459 | *spns3* | 747.26 | 53.32 | 62.15 | 62.07 |
| ENSDARG00000103765 | *stpg1* | 1017.73 | 337.68 | 148.46 | 426.82 |
| ENSDARG00000045408 | *tagln* | 3829.32 | 550.02 | 201.98 | 111.72 |
| ENSDARG00000041734 | *tbc1d32* | 1965.97 | 795.10 | 418.63 | 836.46 |
| ENSDARG00000104125 | *ttc6* | 4166.07 | 1759.51 | 788.07 | 1935.51 |
| ENSDARG00000110627 | *zgc:171310* | 196.70 | 46.77 | 25.89 | 43.92 |
| ENSDARG00000110008 | *znf1004* | 70.56 | 7.48 | 3.45 | 7.64 |
